# Deposition of NiO Nanoparticles on Nanosized Zeolite NaY for Production of Biofuel via Hydrogen-Free Deoxygenation

**DOI:** 10.3390/ma13143104

**Published:** 2020-07-11

**Authors:** Min-Yee Choo, Lee Eng Oi, T. Jean Daou, Tau Chuan Ling, Yu-Chuan Lin, Gabriele Centi, Eng-Poh Ng, Joon Ching Juan

**Affiliations:** 1 Nanotechnology and Catalysis Research Center (NANOCAT), University of Malaya, Kuala Lumpur 50603, Malaysia; alexandriachoo@gmail.com (M.-Y.C.); oi.leann@gmail.com (L.E.O.); 2 Institute of Biological Sciences, Faculty of Science, University of Malaya, Kuala Lumpur 50603, Malaysia; tcling@um.edu.my; 3 School of Chemical Sciences, Universiti Sains Malaysia (USM), Penang 11800, Malaysia; 4 Institut de Science de Matériaux de Mulhouse UMR, Université de Haute-Alsace, Université de Strasbourg, Axe Matériaux à Porosités Contrôlées, 7361, ENSCMu, 3b rue Alfred Werner, 68093 Mulhouse, France; jean.daou@uha.fr; 5 Department of Chemical Engineering, National Cheng Kung University, No. 1 University Road, Tainan 70101, Taiwan; yclin768@mail.ncku.edu.tw; 6 Departments ChiBioFarAm and MIFT, ERIC aisbl and CASPE/INSTM, University of Messina, viale F. Stagno d’Alcontres 31, 98166 Messina, Italy; centi@unime.it; 7 Sunway Campus, Monash University, Jalan Lagoon Selatan, Bandar Sunway 46150, Selangor, Malaysia

**Keywords:** NiO, zeolite Y, deposition–precipitation, triolein, green diesel

## Abstract

Nickel-based catalysts play an important role in the hydrogen-free deoxygenation for the production of biofuel. The yield and quality of the biofuel are critically affected by the physicochemical properties of NiO supported on nanosized zeolite Y (Y65, crystal size of 65 nm). Therefore, 10 wt% NiO supported on Y65 synthesized by using impregnation (IM) and deposition–precipitation (DP) methods were investigated. It was found that preparation methods have a significant effect on the deoxygenation of triolein. The initial rate of the DP method (14.8 g_oil_·h^−1^) was 1.5 times higher than that of the IM method (9.6 g_oil_·h^−1^). The DP-Y65 showed the best deoxygenation performance with a 80.0% conversion and a diesel selectivity of 93.7% at 380 °C within 1 h. The outstanding performance from the DP method was due to the smaller NiO particle size (3.57 ± 0.40 nm), high accessibility (H.F value of 0.084), and a higher Brönsted to Lewis acidity (B/L) ratio (0.29), which has improved the accessibility and deoxygenation ability of the catalyst. The NH_4_^+^ released from the decomposition of the urea during the DP process increased the B/L ratio of zeolite NaY. As a result, the pretreatment to convert Na-zeolite to H-zeolite in a conventional zeolite synthesis can be avoided. In this regard, the DP method offers a one-pot synthesis to produce smaller NiO-supported nanosized zeolite NaY with a high B/L ratio, and it managed to produce a higher yield with selectivity towards green diesel *via* deoxygenation under a hydrogen-free condition.

## 1. Introduction

Currently, fossil fuels (coal, petroleum, and natural gas) are the main energy sources that supply our energy demand. According to the International Energy Agency (IEA), fossil fuels have contributed to nearly 70% of the global primary energy demand where transportation has accounted for 25.8% of the energy used from fossil fuels [1,2]. Burning fossil fuel leads to global warming due to the emission of greenhouse gases (GHG) such as CO_2_ [3]. The ability of the oil crops to utilize the CO_2_ through photosynthesis has made biofuel a carbon neutral fuel [4]. Therefore, biofuel, which is environmentally friendly, renewable, and sustainable, is a promising alternative to substitute fossil fuel [5].

There are different types of feedstock for biofuel production. For example, the first-generation feedstock uses edible oil crops such as palm, soybean and sunflower [6,7]. However, the low productivity and requirement of arable land have received some debates in the scientific community. In the second-generation biofuel feedstock, waste feedstocks such as waste cooking oil and lignocellulosic feedstocks are used, and they are better than the first-generation feedstock in terms of the impact to the environment. However, their supplies to fulfil the large biofuel market is a real challenge [8]. Microalgae has emerged as a third-generation feedstock due to its simple growth requirement and high biomass growth. However, high biomass growth is often accompanied with low oil productivity and vice versa [9]. Therefore, engineered microalgae is considered as the fourth-generation feedstock due to its high CO_2_ sequestration ability, high growth, and lipid productivity [5].

In biofuel production, biodiesel or fatty acid methyl ester (FAME) is derived from the transesterification of triglyceride in oil crops, such as microalgae, soybean, rapeseed, and palm oil, which have been extensively studied [6,10,11]. However, the oxygen content, fluid density, flash point, and viscosity of biodiesel in a high extent have limited its direct application in transportation [12,13]. Hence, it is often blended with petroleum diesel with a low blending ratio (<20%) [12]. As a result, biofuel such as biodiesel only contributes to about 5% of the energy source in the U.S. transportation sector [2]. In order to improve the biofuel quality, the elimination of oxygen atoms from the biofuel is needed so that its performance is comparable with the petroleum-based fuel. Deoxygenation is a promising thermochemical process that enables the removal of oxygenated compounds present in the biofuels. In general, deoxygenation proceeds through two major routes, namely (a) hydrodeoxygenation (HDO) and (b) decarbonylation and decarboxylation (DCO). HDO is a highly efficient process in removing oxygenated compounds but it requires high hydrogen (H_2_) pressure during the process [14]. This becomes a major drawback of the HDO process as the hydrogen gas is mainly derived from the fossil fuels in the current stage [15]. Hence, the production of renewable biofuel from triglycerides without using an external hydrogen source is interesting. For example, DCO reactions using inexpensive solid acid or base catalysts to produce hydrocarbon-like biofuel under H_2_-free condition are reported [16,17,18]. Oi et al. reported that 76.9% of triolein conversions with 32.0% DCO selectivity have been attained over an acidic mesoporous TiO_2_ at 380 °C for 8 h [18]. In a study by Ooi et al., a similar conversion (76.9%) was attained with shorter reaction time (4 h) over 0.2Ti-0.8Al mixed oxide but with a lower DCO selectivity (27.3%) [17]. The performance of the deoxygenation catalysts can be improved by adding active metal species. Metal-supported catalysts with noble metals (Pd, Ru, Pt) and transition metals (Ni, Fe, Cu, Co), have been reported in the deoxygenation reaction [19,20,21,22,23]. However, noble metals are very expensive with limited resources. Therefore, transition metals with a high abundancy and low cost seem to be a better choice as deoxygenation catalysts.

Among the commonly used transition metals (Ni, Fe, Cu, Co), Ni is widely studied in deoxygenation due to its high hydrogenolysis ability [24,25,26]. Besides the selection of active metals, the metal preparation method could affect the physicochemical properties and the reactivity as well [27,28]. There are many synthesis methods in preparing metal-supported catalysts such as impregnation, ion-exchange, sol-gel, and deposition-precipitation. However, each method has its strength and weakness. For instance, the sol-gel (SG) method is capable of achieving good metal dispersion, but it is not suitable for high metal loading as the particles tend to agglomerate into large particles [29]. Meanwhile, ion-exchange (IE) method is also capable of achieving good metal dispersion, but it is limited to the exchange ability of the support. Furthermore, multiple ion-exchange is often needed to achieve high metal loading [30]. On the other hand, despite large metal particle sizes being often produced, the impregnation (IM) method is widely used due to its simplicity, and it is able to achieve high metal loading [31]. Deposition–precipitation (DP) is well known for producing small-sized metal particles [27]. This method involves the precipitation of metal in the form of metal hydroxide followed by deposition on the catalyst. Unlike the previous two methods, the DP method is suitable in preparing metal-supported catalysts with higher metal loadings (~40%) [32]. The variation in the preparation method will lead to different physicochemical properties and lead to a different catalytic performance. For instance, the size of Ni nanoparticles supported on zeolite Beta (H-BEA) has demonstrated a significant difference in the HDO of microalgae oil, where small Ni nanoparticles (particle size ~3.9 nm) prepared using the DP method exhibit a higher reactivity and faster initial rate than Ni particles prepared using the IM method (particle size ~24.7 nm) [27]. However, metallic Ni requires prereduction in a hydrogen atmosphere and is prone to oxidation during treatment. Therefore, metallic metal is not suitable for the H_2_-free reaction environment.

In recent studies, transition metals in oxide form (TMO) have received much interest in hydrogen-free deoxygenation because they do not require a prereduction step [33,34]. In our previous contribution, we tested the deoxygenation ability of various TMOs (Ni, Cu, Co, Mn, and Zn) impregnated on commercial micron-sized zeolite Y in the hydrogen-free deoxygenation of triolein. Among these TMOs, nickel oxide (NiO) showed the best deoxygenation performance [26]. Similarly, the enhancement of the deoxygenation of vegetable oil were also reported for NiO impregnated on hexagonal mesoporous silica (HMS) and aluminum-Santa Barbara Amorphous type material number 15 (Al-SBA-15) [35,36]. However, there is a lack of studies on the effect of NiO particle size towards deoxygenation of nonedible oil. Furthermore, although the DP method has been extensively studied with H- or NH_4_-form zeolite [31,37], the effect of this method in the physicochemical properties such as acidity on Na-form zeolite remains unknown. In conventional zeolite Y synthesis, an H-form zeolite is prepared by the ion-exchange of Na-form zeolite with NH_4_^+^ and followed by calcination [38]. Therefore, the NH_4_^+^ released through the decomposition of urea may serve as the ion-exchange agent, and a two-step calcination can be avoided.

Previously, we reported that the nanosized zeolite NaY could improve the deoxygenation performance of triolein in the absence of a hydrogen source [39]. In this study, we extend the utilization of nanosized zeolite NaY by loading with NiO nanoparticles prepared using different synthesis methods. Due to the high metal content (10 wt% Ni) being used in this study, impregnation (IM) and deposition–precipitation (DP) methods are selected for the investigation. Their physicochemical properties and performances in the H_2_-free deoxygenation of triolein are also discussed.

## 2. Experimental Section

### 2.1. Synthesis of Parent Nanosized Zeolite NaY with Crystal Size of 65 nm (Y65)

The parent Y65 was synthesized according to our previous work [39]. Briefly, solution A was prepared by dissolving NaOH (1.500 g, 99%, Merck, Darmstadt, Germany) and sodium aluminate (2.693 g, 53.0% Al_2_O_3_, 42.5% Na_2_O, Sigma-Aldrich, Malaysia) in distilled water (13.000 g). Solution B was prepared by mixing sodium silicate (45.343 g, 26.5% SiO_2_, 10% Na_2_O, Sigma-Aldrich, Darmstadt, Germany) with NaOH (3.671 g) and distilled water (16.079 g). Solution A was added dropwise to solution B under vigorous stirring in an ice bath. The resulting clear suspension with a molar composition of 8Na_2_O: 0.7Al_2_O_3_: 10SiO_2_: 160H_2_O was aged (25 °C, 24 h) before being subjected to crystallization (120 °C, 70 min). The crystallized solid was collected using high-speed centrifugation (10,000 rpm, 30 min, Heraeus Multifuge X3F Centrifuge, Thermo Fisher Scientific, Waltham, MA, USA) and washed with distilled water until pH 7 before drying at 70 °C overnight.

### 2.2. Preparation of NiO-Supported Y65

#### 2.2.1. Impregnation (IM) Method

Ni(NO_3_)_2_·6H_2_O (1.011 g, 10 wt% Ni metal basis (99%), Merck, Germany) was first dissolved in distilled water (5 mL) before the parent Y65 (2.000 g) was added to the solution. The mixture was continuously stirred for 30 min at ambient temperature. While stirring, the solution was then heated and maintained at 80 °C to evaporate the water. The resulting solid product was dried (70 °C, 15 h), ground into fine powder, and calcined (550 °C, 4 h, 1 °C/min) to produce the IM-Y65 catalyst, which is gray in color.

#### 2.2.2. Deposition–Precipitation (DP) Method with Urea

Typically, an aqueous solution (40 mL) containing Ni(NO_3_)_2_·6H_2_O (1.011 g, 10 wt% Ni metal basis) was first prepared, and 30 mL of the solution was used to make a suspension with parent Y65 (2.000 g). Urea (2.2741 g, 98%, Sigma-Aldrich, Germany), which was equivalent to the Urea:Ni molar ratio of 10:1, was dissolved in the remaining Ni(NO_3_)_2_ solution. The mixture solution was then added dropwise into the zeolite suspension at 70 °C before the DP process was initiated at 90 °C. After 2 h of DP treatment, the suspension was centrifuged (10,000 rpm, 10 min), washed with distilled water until pH 7, oven dried (70 °C, 15 h), ground into fine powder and calcined (550 °C, 4 h, 1 °C/min) to produce the DP-Y65 catalyst, which is light gray in color.

### 2.3. Catalyst Characterization

The X-ray diffraction (XRD) patterns of the zeolites were recorded using a XRD diffractometer (Empyrean, Malvern PANalytical, UK) (Cu Kα, λ = 1.5406 Å, 2θ = 5–50°, 1.5 s per step). The crystallite size of nickel oxide was estimated using the Scherrer equation (Equation (1)):

(1) $$d=\frac{K\lambda }{\beta \;\cos\;\theta }$$

where K is a dimensionless constant, taken as 0.9; λ is the wavelength of X-ray radiation; β is the full width at half-maximum (FWHM); and θ is the diffraction angle. The high resolution transmission electron microscopy (HRTEM) images of the samples were captured with a FEI Tecnai F20 HRTEM microscope (USA) with an accelerating voltage of 200 kV. The porosity of the solids was determined by a nitrogen adsorption analyzer (ASAP 2020, Micromeritics, Norcross, GA, USA). Prior to the analysis, the samples were first degassed under a vacuum (300 °C, 8 h). The pore size distribution of the samples in micropore (0.4 to 1.0 nm) and mesopore (2 to 50 nm) ranges was determined by the density functional theory (DFT) method. The hierarchical factor (H.F) was calculated by using the following equation (Equation (2) [40]:

(2) $$H.F=\frac{V_{\mathrm{mic}}\times S_{\mathrm{ext}}}{V_{\mathrm{tot}}\times S_{\mathrm{BET}}}$$

where V_mic_ is the micropore surface area, S_ext_ is the external surface area, V_tot_ is the total pore volume, and S_BET_ is the Brunauer-Emmett-Telle (BET) surface area. The chemical composition of the zeolites was determined by using a inductively coupled plasma—optical emission spectrophotometry (ICP-OES) spectrometer (Vista MPX, Varian, Atlanta, GA, USA). The Ni content of the samples were determined by an energy dispersive X-ray (EDX) spectrometer (X-max, Oxford Instruments, UK) with an excitation energy of 15 kV. The acidity of the samples was determined by using the temperature programmed desorption-ammonia (TPD-NH_3_) technique with TPD analyzer (TPD/R/O 1100, Thermo Finnigan, USA). The sample (50 mg) was first treated (250 °C, 30 min) under a N_2_ gas flow, followed by saturation with NH_3_ gas (25 °C, 1 h). The excess NH_3_ was subsequently flushed away with N_2_ gas flow (20 mL/min, 30 min). The acidity profile was recorded by a thermal conductivity detector (TCD) detector under helium gas flow (30 mL/min) from 50 °C to 600 °C with a heating rate of 10 °C/min and held for 30 min. The pyridine Fourier transform-infrared spectroscopy (FTIR) spectra were recorded using a FTIR spectrometer (Nicolet 2000, Thermo Fisher Scientific, Waltham, MA, USA). A self-supported wafer (area 2 cm^2^, mass of 11–12 mg) was first prepared before it was inserted into the infrared (IR) cell and degassed under vacuum (10^−3^ mbar, 400 °C, 5 h). The reference spectrum was first recorded after cooling before pyridine (containing 73 ppm H_2_O according to Karl Fischer titration) was introduced to the samples (25 °C, 30 min). The sample was evacuated at 200 °C for 30 min to remove weakly bound pyridine molecules. The concentrations of the Brönsted (C_Brönsted_) and Lewis (C_Lewis_) acid sites were calculated using molar integral extinction coefficients of ε_Brönsted_ = 0.73 cm/µmol and ε_Lewis_ = 0.96 cm/µmol [27]. The coke deposition of the samples was determined by an thermogravimetic analysis (TGA) analysis (STA6000, Perkin Elmer, USA) from 30 to 750 °C with a heating ramp of 10 °C/min and under air condition.

### 2.4. Catalytic Deoxygenation of Triolein

Initially, triolein (10.000 g, glyceryl trioleate, 65%, Sigma-Aldrich, Germany) and a catalyst (0.500 g) were added into a 50 mL semi-batch quartz reactor equipped with a mechanical stirrer, a temperature controller, and a vacuum system. An ice-cold distillation system (4 °C) was used to control the condensation of deoxygenated products. The reaction was performed at 380 °C for 0.15 to 4 h with a catalyst loading of 1 to 9 wt% under stirring (400 rpm) and partial vacuum (10 mbar) conditions without any gas supply. The experiment was performed in triplicates to verify the reproducibility of the reaction.

### 2.5. Analysis of Deoxygenated Products

The fractions of deoxygenated products were analyzed by a Gas Chromatography-Mass Spectrometry (GC-MS) spectrometer (QP2010 Plus, Shimadzu, Kyoto, Japan) with a RTX-5MS column (30.0 m × 0.25 µm × 0.25 mm). All samples were diluted to 500 ppm with *n*-hexane (Merck) and were spiked with 50 ppm of 1-bromohexane (Merck) as internal standard. The diluted samples (1 μL, splitless) were then injected into the GC-MS where the initial oven temperature (40 °C) was raised to 300 °C (5 °C/min) and held at 300 °C for 40 min. The mass spectra were compared with the National Institute of Standards and Testing (NIST) library and standard hydrocarbon solutions of C_8_ to C_20_ (Sigma-Aldrich, Malaysia). Meanwhile, the product distribution of deoxygenated liquids was determined by calculating the peak area of the Gas Chromatography (GC) chromatographs. The conversions of triolein (Equation (3)), organic liquid product (OLP) selectivity (Equation (4)), and hydrocarbon selectivity (Equation (5)) were calculated using the following equations, respectively:

(3) $$\mathrm{Conversion}\;(\%)=\frac{{\mathrm{Mass}\;\mathrm{of}\;\mathrm{reactant}\;}_{\mathrm{initial}}-{\;\mathrm{Mass}\;\mathrm{of}\;\mathrm{reactant}\;}_{\mathrm{final}}}{{\mathrm{Mass}\;\mathrm{of}\;\mathrm{reactant}\;}_{\mathrm{initial}}}\times 100\%$$

(4) $$\mathrm{OLP}\;\mathrm{selectivity}\;(\%)=\frac{\mathrm{Total}\;\mathrm{Area}\;\mathrm{of}\;\mathrm{Desired}\;\mathrm{Organic}\;\mathrm{Product}}{\mathrm{Total}\;\mathrm{Area}\;\mathrm{of}\;\mathrm{the}\;\mathrm{liquid}\;\mathrm{product}\;}\times 100\%$$

(5) $$\mathrm{Hydrocarbon}\;\mathrm{selectivity}\;(\%)=\frac{\mathrm{Area}\;\mathrm{of}\;\mathrm{Hydrocarbon}\;\mathrm{Fraction}}{\mathrm{Total}\;\mathrm{Area}\;\mathrm{of}\;\mathrm{Hydrocarbon}\;C8-C24}\times 100\%$$

The initial rate (g_oil_·h^−1^) was calculated based on the conversion of triolein for the first 30 min.

## 3. Results and Discussion

### 3.1. Catalyst Characterization

#### 3.1.1. X-ray Diffraction (XRD) Analysis

**Figure 1:**
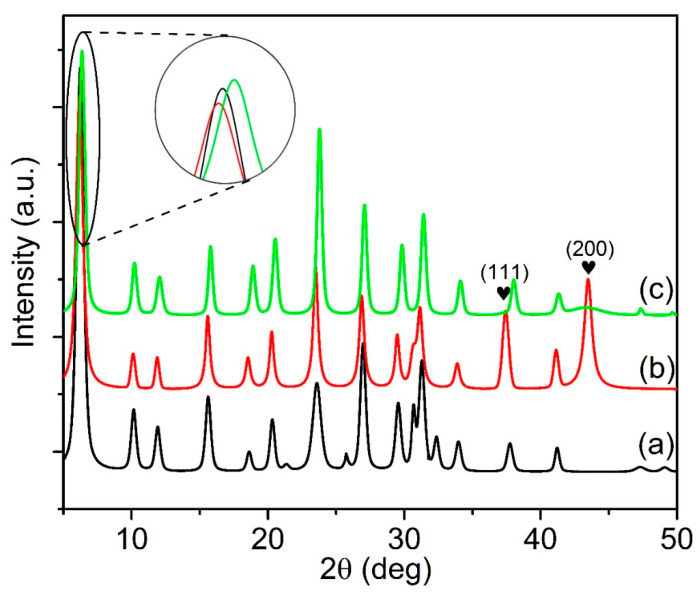
XRD diffractograms of (**a**) Y65, (**b**) IM-Y65, and (**c**) DP-Y65. The symbol indicates the characteristic diffraction peak for nickel oxide.

The XRD diffractograms of parent Y65 and the supported NiO catalysts are shown in Figure 1. The parent Y65 shows well-defined diffraction peaks at 2θ = 6.2°, 10.2°, 12.0°, 15.8°, 19.2°, 20.5°, 23.9°, and 27.0° due to the (111), (220), (311), (331), (440), (533), (642), and (733) planes of zeolite Y, respectively (Figure 1a) [41]. Nevertheless, the addition of NiO to the zeolite followed by a calcination treatment slightly decreased the crystallinity (Figure 1b,c). As shown in Table 1, the Si/Al ratio of Y65 was 1.94 and was slightly altered after loading with NiO. The Si/Al ratio of the IM-Y65 decreased to 1.91. Meanwhile, the Si/Al ratio of DP-Y65 increased up to 1.96. This was consistent with the XRD result in which the diffractograms of DP-Y65 (Figure 1c) was slightly right-shifted but IM-Y65 was more left-shifted compared to parent Y65. This is because the addition of NiO tends to slightly change the coordination environment of Si and Al in the zeolite [42]. The effective doping of NiO on Y65 was confirmed by the presence of the diffraction signals at 2θ = 37.3° and 43.3°, which corresponded to the (111) and (200) planes of the face-centered cubic NiO (JCPDS 47-1049), respectively [35]. No other XRD peaks or unknown phases were detected. The peaks of NiO of IM-Y65 prepared using the impregnation method (Figure 1b) were sharper and very intense, indicating large NiO crystallites (13.2 nm) have been formed. On the contrary, the NiO peaks in DP-Y65 were broader and less intense due to the high dispersion of smaller NiO crystallites on Y65 [43,44]. However, it is difficult to estimate the crystallite size from the broad XRD peaks. Hence, HRTEM was employed to study the particle size distribution and dispersion of the NiO nanoparticles.

#### 3.1.2. HRTEM Analysis

**Figure 2:**
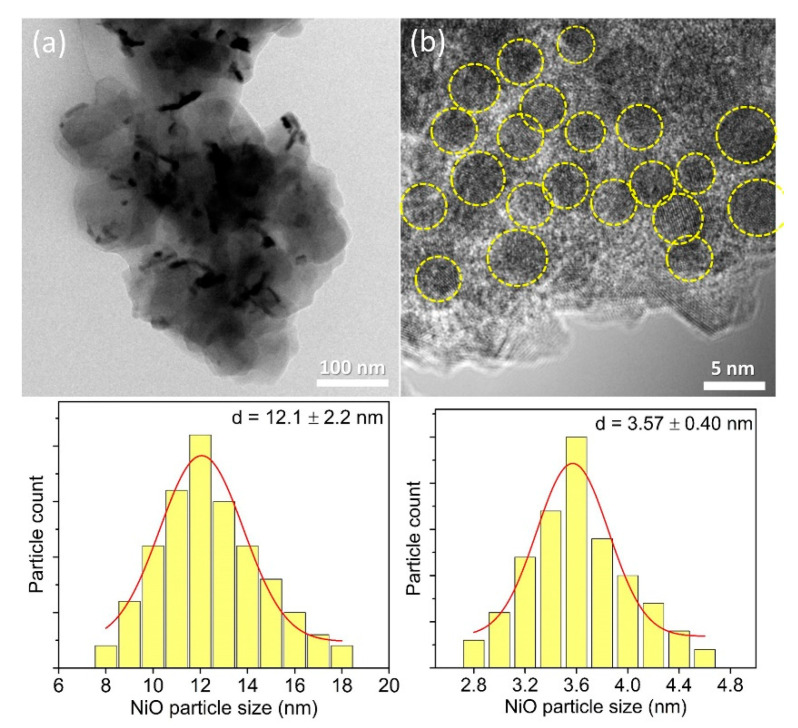
HRTEM images of (**a**) IM-Y65 and (**b**) DP-Y65.

The HRTEM images of the samples are shown in Figure 2. It is shown that the modification approach plays a key role in controlling the particle size and size distribution of NiO active species. The NiO nanoparticles prepared via the IM method were larger, and they tend to aggregate forming large clusters (12.1 ± 2.2 nm) on the surface of Y65 (Figure 2a). For the DP method, NiO nanoparticles with a much smaller size of 3.57 ± 0.40 nm were formed (Figure 2b). In addition, DP methods manage to give a more homogeneous distribution of NiO on the surface of zeolite. These results are in good agreement with the XRD results where IM-Y65 produced larger NiO nanoparticles than DP-Y65 did. The amount of Ni was determined by an EDX analysis. As shown in Table 1, the Ni content in IM-Y65 and DP-Y65 was 9.5 and 10.2 wt%, respectively, which were close to the theoretical Ni content of 10 wt%. It should be borne in mind that the control of the particle size of NiO is important as it affects the overall physicochemical properties of the solid where the particle size normally is governed by the synthesis conditions. In order to ensure the generation of NiO particles in the nanometer range, the use of a weak base (e.g., urea) instead of a strong base (e.g., NaOH) is important, as urea can slowly release the hydroxide ions without an abrupt change in pH value (6, 7).

#### 3.1.3. N_2_ Sorption Analysis

**Figure 3:**
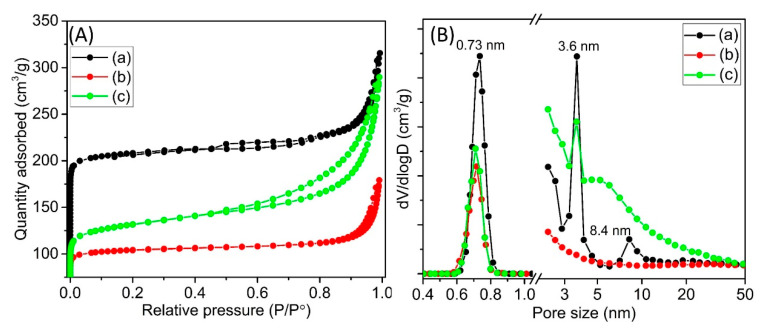
(**A**) N_2_ sorption isotherms and (**B**) pore size distribution of (a) Y65, (b) IM-Y65, and (c) DP-Y65.

The addition of NiO nanoparticles on the zeolite support might influence the surface porous properties, and hence an N_2_ sorption isotherm analysis is performed. The parent Y65 shows a combination of types I and IV isotherm and a large H4-type hysteresis at high P/P_o_ due to the presence of intercrystal mesoporosity ((a) in Figure 3A) [45]. The mesoporosity appeared due to the crystal packing of nanosized zeolite crystals [46]. It possessed a S_BET_ and a V_tot_ of 661 m^2^/g and 0.49 cm^3^/g, respectively. When the NiO nanoparticles are formed on the zeolite support by means of various methods, the IM-Y65 and DP-Y65 still exhibit type I and IV isotherms but with lower N_2_ uptake ((b, c) in Figure 3A). As shown by the pore size distribution in the micropore region (0.4–1.0 nm), the parent Y65 possessed a micropore size of 0.73 nm. The micropore size slightly decreased to about 0.70 nm with lower intensity after the modification. This observation indicates that some micropores (especially IM-Y65) were occupied by the Ni^2+^ ions through the ion-exchange aside from the partial dissolution of the zeolite framework as shown by the decrease in zeolite crystallinity after the modifications [14,35] ((b, c) in Figure 3A). As a result, lower values of S_BET_, S_mic_, V_mic_ and V_tot_ were recorded. Nevertheless, the S_ext_ of DP-Y65 increased nearly one-fold (from 62 to 124 m^2^/g), but the IM-Y65 experienced a slight decrease in the S_ext_ (43 m^2^/g). The difference in S_ext_ observed in these samples can be explained by the different particle sizes of the NiO nanoparticles distributed on the zeolite surface where smaller NiO particles have a higher surface area than the larger one [28]. The NiO addition also affect the molecular diffusion properties of the supported catalysts, and hence the hierarchical factor (H.F) of the zeolite samples was calculated. As seen, the H.F value of IM-Y65 slightly decreased from 0.056 to 0.052 when it was modified with NiO impregnation (Table 2). Interestingly, the H.F value increased to 0.084 for DP-Y65, implying that the diffusion of bulky molecules such as triolein in this catalyst is significantly improved.

In terms of mesoporosity, the parent Y65 contains two different mesopores of 3.6 and 8.4 nm. However, these mesopores disappear in the IM-Y65 sample, which clearly indicates that the large NiO particles prepared by the IM method not only occupy the micropores, but they also covered the intercrystal mesopores of the zeolite. For DP-Y65, the number of the 3.6 nm mesopore was reduced while the mesopore of 8.4 nm disappeared and subsequently a new mesopore size shouldered at 5.0 nm was formed. This result suggests that some NiO nanoparticles (ca. 3.6 nm) have entered the large mesopore (8.4 nm) and therefore result in the shrinkage of the mesopore to 5.0 nm. The pore size distribution analysis is in good agreement with the results reported by Cheng et al., where the mesopore diameter was slightly decreased after loading with metal nanoparticles [47].

#### 3.1.4. TPD-NH_3_ and Pyridine-FTIR Analysis

**Figure 4:**
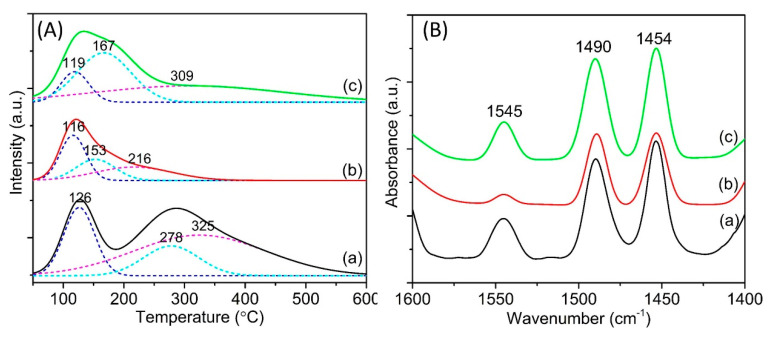
(**A**) TPD-NH_3_ profiles and (**B**) pyridine-FTIR spectra of (a) Parent Y65, (b) IM-Y65, and (c) DP-Y65.

Figure 4A shows the TPD-NH_3_ profiles of the parent and NiO-modified Y65 zeolite samples. The deconvolution of the TPD-NH_3_ profiles give rise to the acid sites with different acidity strengths. As shown, parent Y65 exhibited three deconvoluted peaks at 126, 278, and 325 °C which corresponded to the weak, medium, and medium-strong acid sites ((a) in Figure 4A). When the NiO nanoparticles were supported on the Y65 using different approaches, a significant change in the acid strength was observed in the resulting NiO-modified Y65. As discussed earlier, the change in the Si/Al ratio is minimal, and therefore the change in acidity profile was primarily due to the NiO modifications. The acid strength was reduced across the NiO-modified Y65 as the maximum of medium strength acidities at 278 °C was shifted to the range of 153 and 167 °C. At the same time, the maximum of medium-strong acidity (325 °C) was shifted to a lower desorption temperature (216–309 °C) as well. Only the DP-Y65 retained the medium-strong acidity of parent Y65, although the desorption temperatures are slightly lower (309 and 325 °C, respectively). This is due to the fact that some of the acid sites of zeolite Y have been covered by NiO nanoparticles [20]. The amount of the medium-strong IM-Y65 was 1.14 mmol/g (Table 2). This value was significantly lower than those of parent Y65 and DP-Y65 (2.70 and 2.10 mmol/g, respectively). Nevertheless, the amount of medium strength acidity has increased from 0.82 mmol/g (parent Y65) to the range of 0.92–2.32 mmol/g after supporting with NiO nanoparticles, revealing that NiO introduces medium strength acidity onto the zeolite support.

The distribution of Brönsted and Lewis acid sites in the samples were studied by using pyridine-FTIR and is shown in Figure 4B. The IR bands located at 1545, 1490, and 1454 cm^−1^ correspond to the Brönsted acid sites (B), the total acidity (B+L), and the Lewis acid sites (L), respectively [48]. When the NiO nanoparticles were introduced onto the parent Y65 by impregnation (IM-Y65), the Brönsted acidity has decreased significantly whereas DP-Y65 still shows considerably high peak intensity. The reduction in Brönsted acidity is expected due to the loss in zeolite crystallinity (as shown by the XRD and BET results) and the occupying of NiO on the Brönsted acid sites. On the other hand, the Lewis acidity increased with the addition of NiO nanoparticles, which is in line with the observation reported in Ref. [49]. The pyridine-FTIR data are also tabulated in Table 2. DP-Y65 has the highest B/L ratio (0.29) while the IM-Y65 has a relatively lower B/L ratio (0.08). The high Brönsted acidity of DP-Y65 can be due to the partial decomposition of the NH_4_^+^ ion to H^+^ during the synthesis calcination step. The NH_4_^+^ ions were produced from the decomposition of urea (Equation (6)).

(6) $$\mathrm{CO}{\left({\mathrm{NH}}_{2}\right)}_{2}+3H_{2}O\;\to 2{\mathrm{NH}}_{4}{}^{+}+{\mathrm{HCO}}_{3}{}^{-}+{\mathrm{OH}}^{-}$$

The partial exchange of the Na^+^ ion with NH_4_^+^ ion in DP-Y65 can be proven by the band at 1440 cm^−1^, which corresponds to the NH_4_^+^ [50] (see Supporting Information: Figure S1). This signal disappeared after the calcination step, indicating that the NH_4_^+^ has been decomposed into H^+^ and NH_3_. In addition, this claim can also be proven by the decrease seen in the Na/Al ratio of DP-Y65 (0.43) as compared to that of IM-Y65 (0.80), which is shown in Table 1. Hence, the DP method using urea not only is able to produce smaller NiO nanoparticles, but it also introduces the Brönsted acidity to the NaY zeolite.

### 3.2. Catalytic Deoxygenation Activity

**Figure 5:**
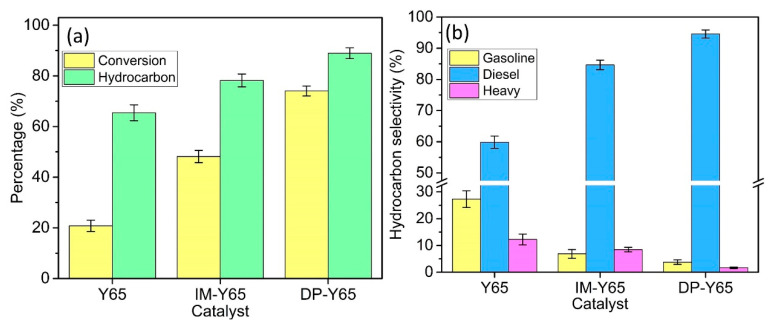
(**a**) Comparison study of conversion and hydrocarbon product and (**b**) hydrocarbon selectivity from deoxygenation of triolein over synthesized catalysts.

The deoxygenation reaction of triolein over the parent Y65 and NiO-modified Y65 prepared by a different synthesis method was determined and is shown in Figure 5. Parent Y65 only shows a 20.8% triolein conversion after reacting at 380 °C for 0.5 h (initial rate: 4.2 g_oil_·h^−1^). The deoxygenation performance of the NiO-modified Y65 was significantly improved as its initial rates were at least doubled than that of parent Y. Specifically, DP-Y65 revealed the highest triolein conversion of 74.0%, equivalent to the initial rate of 14.8 g_oil_·h^−1^. In contrast, IM-Y65 showed a triolein conversion of 46.2% or 9.6 g_oil_·h^−1^. In terms of liquid product distribution, the deoxygenated liquid of parent Y65 contains 65.45% C_8_–C_20_ hydrocarbon. For NiO-modified Y65, DP-Y65 showed a higher C_8_–C_20_ hydrocarbon selectivity (88.9%) than IM-Y65 did (78.2%). Despite having the same amount of Ni content (10 wt%) used in the preparation of NiO, different deoxygenation performances were obtained. This result clearly shows that the deoxygenation of triolein is greatly influenced by the physicochemical properties of the NiO-modified zeolite. DP-Y65 exhibited a smaller NiO particle size (3.57 nm), a larger external surface area (124 m^2^/g), a higher medium and medium-strong acidity with high B/L ratio (0.29), which has facilitated the diffusion of large triolein molecules (~4.4 nm) into the active site and transformed them into deoxygenated products [51]. In contrast, the low external surface area and less medium strength acidity of IM-Y65 shows lower deoxygenation activity.

The 65 wt% triolein mainly consists of C_16_ and C_18_ fatty acids and a trace amount of other C_14_ to C_20_ fatty acids [52]. As we mentioned earlier, the deoxygenation route in the hydrogen-free condition is dominated by the DCO route instead of the HDO route, which requires a high pressure of H_2._ Theoretically, the deoxygenation of triolein in the hydrogen-free condition will produce C_15_ and C_17_ hydrocarbon. However, in the real deoxygenation condition, there are many side reactions that occur, such as cracking and polymerization, which will result in hydrocarbon with different chain lengths. The hydrocarbon can be classified into gasoline, diesel, and heavy hydrocarbon according to their carbon chain length [26,39]. As shown in Figure 5b, the liquid product of parent Y65 contained 27.3% gasoline (C_8_–C_12_), 59.8% of diesel (C_13_–C_18_), and 12.3% of heavy hydrocarbon (C_19+_). The high gasoline content on the parent Y65 may due to the large amount of strong acidity, which facilitates the cracking reaction. The amount of strong acid sites of the NiO-modified Y65 were reduced according to the acidity characterization. As a result, the addition of NiO on the Y65 has greatly improved the diesel selectivity to more than 84.6%. Among the NiO-modified Y65, DP-Y65 showed a higher diesel selectivity (94.6%) than IM-Y65 (84.6%). At the same time, a low selectivity towards gasoline (3.8%) and heavy hydrocarbon (1.7%) on DP-Y65 indicated that the occurrence of cracking and polymerization was very rare. This result is very promising as the polymerization of an organic product will result in the formation of coke, which in turns increase the possibility of catalyst deactivation.

### 3.3. Effect of Catalyst Loading

**Figure 6:**
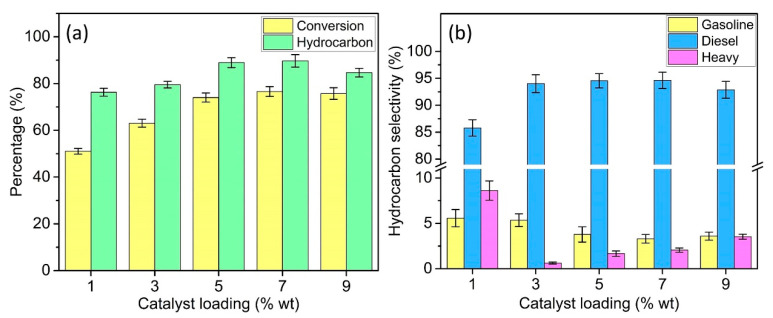
Effect of catalyst loading on (**a**) conversion and hydrocarbon product and (**b**) hydrocarbon selectivity from deoxygenation of triolein over DP-Y65.

Figure 6 shows the conversion, hydrocarbon distribution, and selectivity as a function of catalyst loading (1 to 9 wt%) at 380 °C for 0.5 h. Based on the result, the range of catalysts from 1 to 7 wt% showed improvement in the deoxygenation performance. The triolein conversion gradually increased from 51.1% (1 wt%) to 76.6% (7 wt%). At the same time, the hydrocarbon selectivity also improved from 76.29% (1 wt%) to 89.8% (7 wt%). This improvement is due to the increase of the active site as the catalyst loading increased. In terms of the diesel range hydrocarbon, it progressively increased from 85.8% (1 wt%) to 94.6% (7 wt%) and decreased to 92.8% at the catalyst loading of 9 wt%. The best result was obtained with 7 wt% catalyst loading. The increased catalyst loading to 9 wt% has slightly decreased the performance of deoxygenation. It suggests that an excessive amount of catalyst loading might lead to the occurrence of side reaction such as cracking and polymerization. This can be seen through the slight increase in the gasoline and heavy hydrocarbon at 9 wt% (3.6% and 3.53%, respectively). Therefore, a 7 wt% catalyst loading was the most suitable since it resulted in the optimal triolein conversion (76.6%), hydrocarbon selectivity (89.8%), and diesel range hydrocarbon selectivity (94.6%).

### 3.4. Effect of Reaction Time

**Figure 7:**
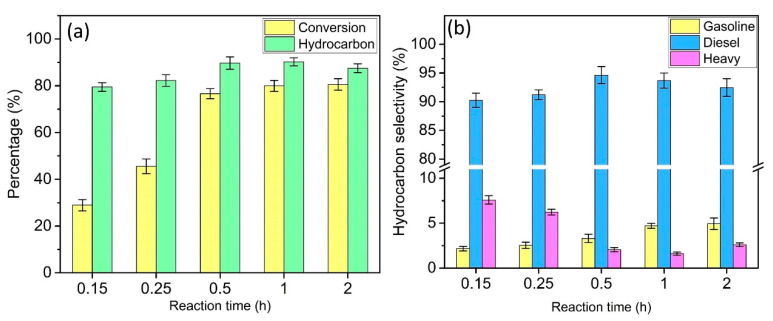
Effect of reaction time on (**a**) conversion and hydrocarbon product and (**b**) hydrocarbon selectivity from deoxygenation of triolein over DP-Y65.

Figure 7 shows the conversion, hydrocarbon distribution, and selectivity as a function of reaction time (0.15 to 2 h) at 380 °C with a catalyst loading of 7 wt%. As shown in Figure 7a, the triolein conversion progressively increased from 29.0% at 0.15 h to 80.0% at 1 h and remained at a plateau at 2 h. At the same time, the hydrocarbon selectivity has increased from 79.5% at 0.15 h to a maximum of 90.3% at 1 h before decreasing to 87.5% at 2 h. In terms of diesel range hydrocarbon selectivity, it increased from 90.3% at 0.15 h to 94.63% at 0.5 h and slightly declined to 92.4% at 2 h. The selectivity for heavy hydrocarbon decreased from 7.6% at 0.15 h to 1.6% at 1 h before increasing to 2.6% at 2 h, while for the gasoline range hydrocarbon, it increased as the reaction time increased. This is due to the longer residence time on the catalyst surface, which could facilitate the decarboxylation reaction. Nevertheless, an excessive long reaction time can lead to product cracking and polymerization, which reduces the amount of diesel range hydrocarbon. Therefore, a 1 h reaction time was the most suitable and economical since it has a relatively high triolein conversion and product selectivity.

### 3.5. Reusability

**Figure 8:**
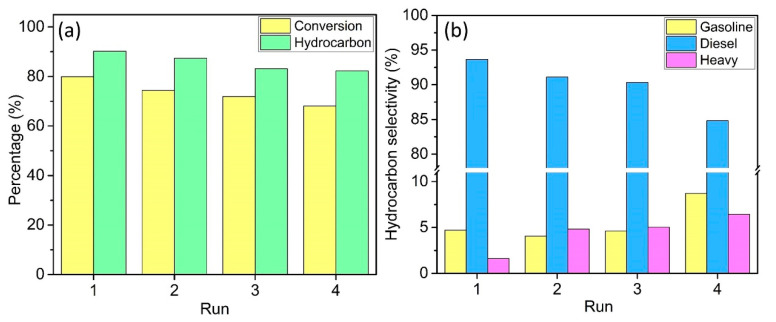
Reusability study of (**a**) conversion and hydrocarbon product and (**b**) hydrocarbon selectivity from deoxygenation of triolein over DP-Y65. Reaction conditions: temperature 380 °C, time 1 h, catalyst loading 7 wt%, pressure 10 mbar, and stirring speed 400 rpm.

Catalyst reusability is a very important aspect in the catalysis as it will reduce the overall production cost if the catalyst has a very long shelf life. The catalyst reusability study of DP-Y65 was further investigated at 380 °C for 1 h with a 7 wt% catalyst loading. As shown in Figure 8, the deoxygenation activity of the DP-Y65 decreased across the consecutive runs. The triolein conversion decreased progressively from 80.0% from the 1st run to 68.1% at the 4th run. In terms of product selectivity, the hydrocarbon selectivity and the diesel range hydrocarbon decreased while the selectivity towards gasoline and heavy hydrocarbon was increased. Based on the XRD result (Figure S2), the recycled catalyst was still showing the same diffraction pattern as the fresh catalyst. This suggests that the recycled catalyst still maintained the framework stability after consecutive reactions. A TGA analysis was carried out to study the extent of coke formation of the catalyst surface. As shown in Figure S3, the fresh DP-Y65 shows a total weight loss about 20.6% while the recycled DP-Y65 shows a total weight loss of about 18.5%. Based on the derivative thermogravimetric (DTG) result, the weight loss of fresh DP-Y65 with a DTG signal at 118 °C corresponds to the physisorbed water or trapped solvent. The recycled DP-Y65 showed two additional DTG signals at 466 and 638 °C, which correspond to the decomposition of coke with different hardnesses [49]. The amount of coke deposited on the DP-Y65 was 14.64% based on the weight loss between 250 and 750 °C. Although the small NiO nanoparticles supported on Y65 is capable of increasing deoxygenation performance, the formation of hard coke with a DTG signal at 638 °C has led to the decreased performance in the reusability studies. This can be due to the reduced mesoporosity after loading with NiO, as evidenced by the N_2_ sorption analysis in previous section. Therefore, it is important for the future studies to include the investigation of mesoporosity in the zeolite to obtain a better catalyst for biofuel production through H_2_-free deoxygenation.

## 4. Conclusions

In conclusion, the 10 wt% of NiO supported on the nanosized Y65 were successfully synthesized with two synthesis methods, namely IM and DP. The modification of the NiO onto zeolite Y has improved the deoxygenation performance of triolein in the absence of hydrogen and solvent conditions. Parent Y65 only showed a 20.8% triolein conversion with a 65.45% hydrocarbon distribution and a 59.8% diesel range hydrocarbon after reacting at 380 °C for 0.5 h with 5 wt% catalyst loading. After loading with 10 wt% of NiO, the deoxygenation activity was significantly improved. The initial rate was at least doubled that that of parent Y65, which were >9.6 g_oil_·h^−1^ and 4.2 g_oil_·h^−1^, respectively. In terms of product selectivity, >78.2% hydrocarbon product and >84.6% diesel range hydrocarbon was attained. However, different synthesis methods have a decisive role in the final deoxygenation performance of NiO supported on Y65. DP-Y65 showed 1.5 times enhancement in the initial rate as compared to IM-Y65, which were 14.8 g_oil_·h^−1^ and 9.6 g_oil_·h^−1^, respectively. The superior performance of the DP-Y65 was due to the synergistic effect of (a) small-sized NiO particles (~3.6 nm), (b) high H.F value (0.084), and (c) high B/L ratio (0.29). Comparing to the IM method, the DP method is able to produce metal oxide with a smaller nanoparticle size and improve the acidity profile of the Na-form zeolite. Thus, the DP method is ideal for the preparation of metal oxide nanoparticles supported on Na-form zeolite with enhanced acidity. Meanwhile, the DP-Y65 showed the highest triolein conversion (80.0%), a 90.3% hydrocarbon distribution, and a 93.7% diesel range hydrocarbon at 380 °C for 1 h with 7 wt% catalyst loading. However, the reusability studies suggest that the deactivation of deoxygenation reaction was due to the deposition of coke. This coke deposition may due to the decrease of mesoporosity after NiO modification, which reduces the diffusion of the heavy hydrocarbon from the zeolite. Therefore, it is important for future studies to include the investigation of mesoporosity in the zeolite in order to obtain a better catalyst for biofuel production through H_2_-free deoxygenation.

## Figures and Tables

**Table 1:** Chemical composition and physical properties of Y65 and NiO-modified Y65.

Samples	S_BET_^a^	S_mic_^b^	S_ext_^c^	V_mic_^d^	V_meso_^e^	V_tot_^f^	Ni Content ^g^	Si/Al Ratio ^h^	Na/Al Ratio ^h^
Y65	661	599	62	0.29	0.20	0.49	-	1.94	1.04
IM-Y65	420	377	43	0.14	0.14	0.28	9.5	1.92	0.80
DP-Y65	503	379	124	0.15	0.29	0.44	10.2	1.96	0.43

^a^ BET surface area (m^2^/g); ^b^ Micropore surface area (m^2^/g); ^c^ External surface area (m^2^/g); ^d^ Micropore volume (cm^3^/g); ^e^ Mesopore volume (cm^3^/g); ^f^ Total pore volume (cm^3^/g); ^g^ Determined by EDX; ^h^ Determined by ICP-OES.

**Table 2:** Surface acidity of Y65 and NiO-modified Y65.

Samples	TPD-NH_3_ Acidity (mmol/g)				Py-FTIR Acidity (mmol/g) at 200 °C				H.F ^a^
	Weak	Medium	Medium-Strong	Total	Brönsted (B)	Lewis (L)	Total Acid (B+L)	B/L Ratio	
Y65	1.09	0.82	2.70	4.62	0.38	1.39	1.77	0.27	0.056
IM-Y65	1.29	0.92	1.14	3.35	0.10	1.18	1.28	0.08	0.052
DP-Y65	0.63	1.94	2.10	4.67	0.50	1.72	2.22	0.29	0.084

^a^ Hierarchical Factor = (V_mic_ × S_ext_)/(V_tot_ × S_BET_).

## Supplementary Materials

The following are available online at https://www.mdpi.com/1996-1944/13/14/3104/s1, Figure S1. FTIR spectra of (a) uncalcined DP-Y65 and (b) calcined DP-Y65.; Figure S2. XRD diffractograms of (a) fresh DP-Y65 and (b) used DP-Y65 after 4th consecutive catalytic reaction runs; Figure S3. TG/DTG thermograms of (a) fresh DP-Y65 and (b) used DP-Y65 after 4th catalytic reaction runs.
