# Supplementary material for: Deposition of NiO Nanoparticles on Nanosized Zeolite NaY for Production of Biofuel via Hydrogen-Free Deoxygenation
Source: Materials (Basel). 2020 Jul 11;13(14):3104. doi: 10.3390/ma13143104 (PMC7412304; doi:10.3390/ma13143104)
Supplement: Supplementary file 1 [file materials-13-03104-s001.pdf]

materials

Supplementary materials

Deposition of NiO Nanoparticles on Nanosized Zeolite NaY for Production of Biofuel via Hydrogen-Free Deoxygenation

Min-Yee Choo [1,2,3], Lee Eng Oi [1], T. Jean Daou [4], Tau Chuan Ling [2], Yu-Chuan Lin [5], Gabriele Centi [6], Eng-Poh Ng [3,*] and Joon Ching Juan [1,7,*]

1 Nanotechnology and Catalysis Research Center (NANOCAT), University of Malaya, 50603 Kuala Lumpur, Malaysia; alexandriachoo@gmail.com (M.-Y.C.); oi.leann@gmail.com (L.E.O.)
2 Institute of Biological Sciences, Faculty of Science, University of Malaya, 50603 Kuala Lumpur, Malaysia; tcling@um.edu.my
3 School of Chemical Sciences, Universiti Sains Malaysia (USM), 11800 Penang, Malaysia
4 Institut de Science de Matériaux de Mulhouse UMR, Université de Haute-Alsace, Université de Strasbourg, Axe Matériaux à Porosités Contrôlées, 7361, ENSCMu, 3b rue Alfred Werner, 68093 Mulhouse, France; jean.daou@uha.fr
5 Department of Chemical Engineering, National Cheng Kung University, No. 1 University Road, Tainan 70101, Taiwan; yclin768@mail.ncku.edu.tw
6 Departments ChiBioFarAm and MIFT, ERIC aisbl and CASPE/INSTM, University of Messina, viale F. Stagno d'Alcontres 31, 98166 Messina, Italy; centi@unime.it
7 Sunway Campus, Monash University, Jalan Lagoon Selatan, 46150 Bandar Sunway, Selangor, Malaysia.
* Correspondence: epng@usm.my (E.-P.N.); jcjuan@um.edu.my (J.C.J.)

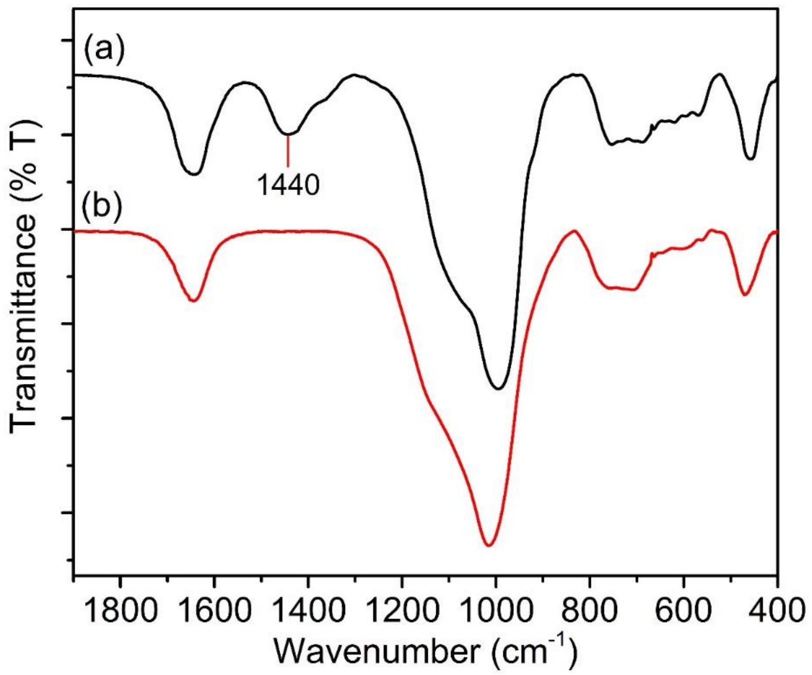

Figure S1. FTIR spectra (**a**) Uncalcined DP-Y65 and (**b**) Calcined DP-Y65.

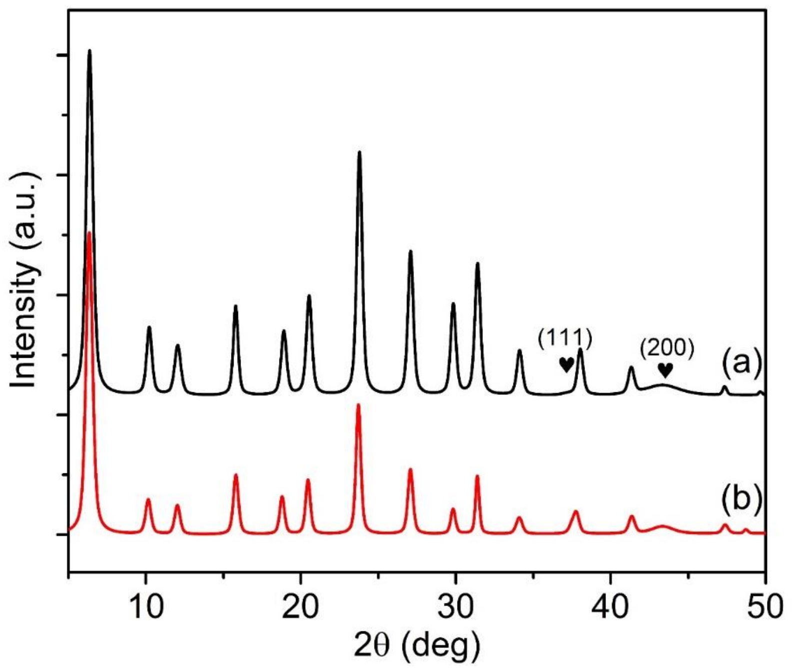

Figure S2. XRD diffractograms of (**a**) Fresh DP-Y65 and (**b**) Used DP-Y65 after 4th catalytic runs.

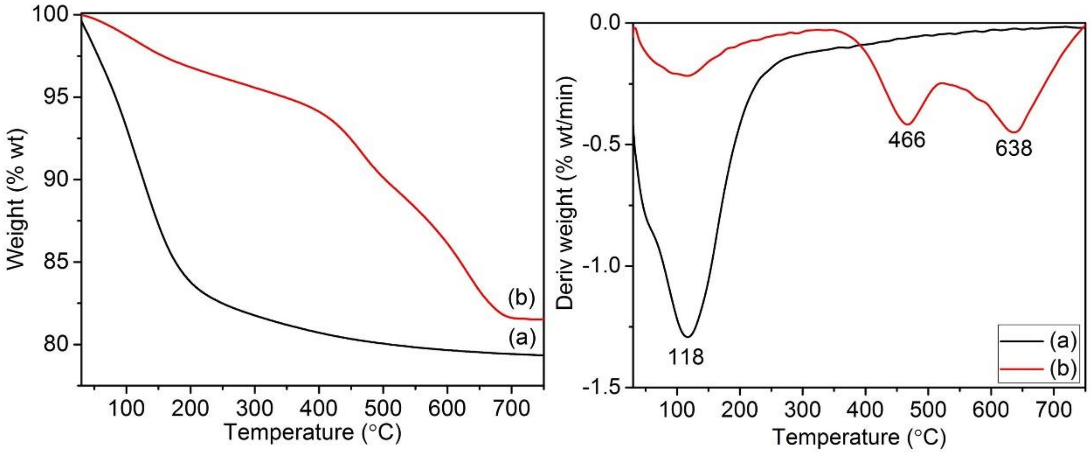

Figure S3. TG/DTG thermograms of (**a**) Fresh DP-Y65 and (**b**) Used DP-Y65 after 4th catalytic runs.

© 2020 by the authors. Licensee MDPI, Basel, Switzerland. This article is an open access article distributed under the terms and conditions of the Creative Commons Attribution (CC BY) license (http://creativecommons.org/licenses/by/4.0/).
